# Automated, longitudinal measures of drinking behavior provide insights into the social hierarchy in dairy cows

**DOI:** 10.3168/jdsc.2023-0487

**Published:** 2023-12-09

**Authors:** Borbala Foris, Bianca Vandresen, Kehan Sheng, Joseph Krahn, Daniel M. Weary, Marina A.G. von Keyserlingk

**Affiliations:** 1 Animal Welfare Program, The University of British Columbia, Vancouver, BC, V6T 1Z6, Canada; 2 Institute of Animal Welfare Science, University of Veterinary Medicine, Vienna, Austria 1210

## Abstract

Dairy cows compete for feed and water access on commercial farms. In this study we used EloSteepness to assess the summed Elo winning probabilities (i.e., dominance) of 87 cows housed in a dynamic group and compared the resulting social hierarchies based on their steepness (i.e., the average degree of differences in winning probability between adjacently ranked individuals in the group, ranging from 0 to 1). We identified a hierarchy at the drinker with a steepness of 0.55 ± 0.02 (SD), whereas the hierarchy detected at the feeder during the same time period was 0.45 ± 0.02, indicating smaller dominance differences among cows when competing for feed compared with competing for water. Individual cows' winning probabilities at the feeder and drinker were moderately correlated (r_s_ = 0.55), and cows at the lower and upper ends of the hierarchy showed good agreement. We compared the drinker hierarchy between hot (i.e., temperature-humidity index [THI] ≥72) and normal (i.e., THI <72) periods. The hierarchy steepness was similar in both hot (0.54 ± 0.03) and normal conditions (0.56 ± 0.03), and there was a strong correlation in cows' individual winning probabilities across these periods (r_s_ = 0.87). Cows with higher winning probability visited the drinker less frequently (hot: r_s_ = −0.40, normal: r_s_ = −0.33) but had a higher average daily water intake (hot: r_s_ = 0.38, normal: r_s_ = 0.37). We also found evidence that individual cows' drinking times differ depending on their winning probability; cows with lower winning probability shifted their drinking times to before or after the visit peak after milking. Automatically identifying cows with consistently high or low winning probabilities using drinkers may help inform grouping decisions and water provision on farms.

## Highlights

• The social hierarchy at drinkers can be automatically assessed in groups of cows.
• Dominance hierarchies at drinkers and feeders were moderately correlated.
• The drinker-based hierarchy was stable between hot and normal temperature periods.
• Cows' dominance influenced when, how often, and how much they drank.

## Graphical Abstract

**Figure:**
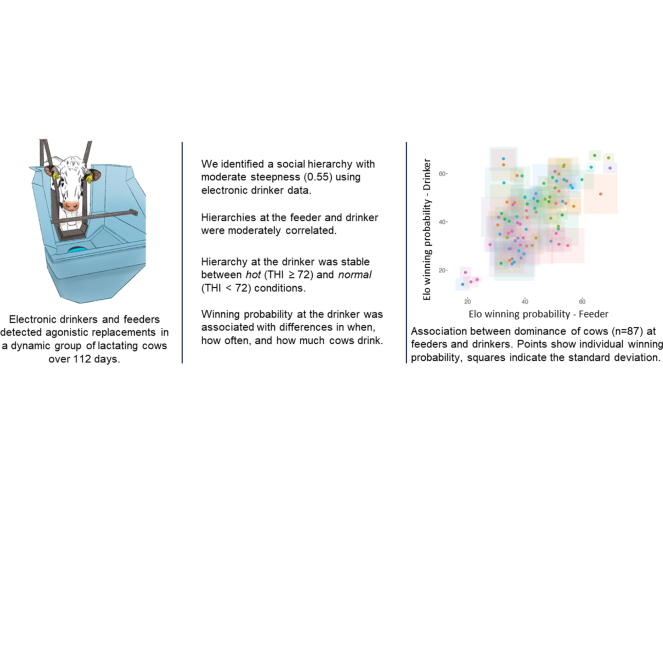
**Summary** Competition for water may affect the welfare and productivity of dairy cows. We investigated whether data from electronic drinkers over a longer time period allowed for the detection of a social hierarchy in lactating cows. Using automatically detected agonistic interactions, we found differences in the winning probabilities among cows, including the identification of a hierarchy with moderate steepness. The hierarchy at the drinker was stable across hot and normal weather periods and showed a positive association with the hierarchy at the feeder. We also found evidence that cows differ in their drinking behavior according to their dominance. More successful cows had fewer visits, higher water intake, and a higher proportion of their daily drinker visits took place during “peak competition” time after milking.

Competitive behavior contributes to the establishment of dominance hierarchies within cattle groups (Wierenga, 1990). One function of an established social hierarchy is to limit aggressive encounters in the group, but the position in the hierarchy can also influence individuals' access to resources (Drews, 1993). Most of the work on competitive behavior in dairy cattle investigated interactions over resources such as feed (Krawczel et al., 2012; Crossley et al., 2017), lying areas (Fregonesi et al., 2007), and the mechanical brush (Reyes et al., 2022). However, competition at the drinker has received less attention (but see Coimbra et al., 2012). Water is an essential resource for dairy cattle and sufficient water intake is important for milk production (Meyer et al., 2004). Changes in climate may place increasing importance on understanding how this resource is used in social groups (Jensen and Vestergaard, 2021). Competition at the drinker has been shown to increase with hot weather, but the effects of increased competition for water on the social hierarchy and individual drinking behavior are not well understood (McDonald et al., 2020).

Much of the work on drinking behavior has relied on either live observation (Pinheiro Machado Filho et al., 2004) or video (Burkhardt et al., 2022), which is labor intensive. Advances in electronic monitoring allow for automated measures of drinking behavior from water bins (e.g., Oliveira et al., 2018). Individual visit data can also be used to detect agonistic replacements (i.e., one cow pushing another away from the bin and occupying her spot within a short time; McDonald et al., 2019).

Previous work has been limited by relatively few agonistic interactions per day at drinkers (Foris et al., 2019; McDonald et al., 2020); however, multiday observations may provide sufficient data (10 to 20 interactions per animal in the group; Sánchez-Tójar et al., 2018) for developing reliable estimates of the social hierarchy at the drinker. Many dominance calculation methods require stable groups or measure overall competitive success without considering variation among the interaction partners of cows. The EloSteepness method (Neumann and Fischer, 2022) is a dynamic dominance assessment method based on Elo rating (Neumann et al., 2011) that overcomes these challenges and is preferred for measuring the social hierarchy within dynamic groups over a longer period.

The steepness of a social hierarchy ranges from 0 to 1 and it reflects the average degree of differences in the dominance scores of every adjacently ranked individual. Higher steepness indicates more authoritarian (i.e., despotic) and lower steepness more egalitarian social groups (de Vries et al., 2006). Methods for steepness calculation have recently been improved to reduce bias from unobserved relationships and to incorporate the uncertainty of measurements (i.e., EloSteepness; Neumann and Fischer, 2022). To our knowledge, EloSteepness has not been used to characterize cattle competition behavior at the drinker.

Agonistic interactions can be influenced by differences in how individuals value resources (Dehnen et al., 2022). Thus, dominance hierarchies based on drinker replacements might differ from those at other resources (Hand, 1986). Replacements at the feeders have been used to estimate dominance hierarchies (Foris et al., 2019), but the relationship between feed-based and water-based hierarchies is unclear.

Individual motivation to access resources and engage in agonistic interactions can vary with factors such as changes in resource quality (feed; Hosseinkhani et al., 2008) or temperature-humidity index (**THI**; water; Stoner et al., 2006), affecting dominance hierarchies. Elevated THI could also increase competition for water (McDonald et al., 2020), but to our knowledge no work has investigated if an increase in THI leads to a change in the water-based dominance hierarchy.

In our previous work focusing on transition cows (McDonald et al., 2020), individuals were classified based on their competitive success at the drinker (low, middle, high). We noted no differences between these categories in daily drinker visit duration or frequency. However, in an exploratory analysis when the THI was >72, cows with low competitive success shifted drinking from the afternoon peak competition period to later in the evening.

In the current study, we build upon our previous work and recent advances in dominance calculation methodology to (1) determine if automated longitudinal measures of agonistic interactions at drinkers allow for the estimation of a social hierarchy in dynamic lactating dairy cow groups, (2) compare individual cows' position in the drinker-based and feeder-based hierarchy, (3) compare the drinker-based hierarchy steepness under different THI conditions, and (4) investigate the association between social dominance and individual drinking behavior under different THI conditions.

We collected data from 87 lactating dairy cows (mean ± SD parity of 3.1 ± 1.3; DIM of 204.6 ± 54.26) at The University of British Columbia (**UBC**) Dairy Education and Research Centre. Cows were housed in a dynamic group, the size of which was kept at 48 animals with access to 48 sand-bedded lying stalls, 30 electronic feed bins, and 5 electronic water bins (Chapinal et al., 2007; Insentec RIC, Hokofarm group, the Netherlands). Bins were installed in a row and all cows were able to access all bins. Each bin could only be accessed by one cow at a time. All procedures were approved by the UBC Animal Ethics Committee (protocol number A19–0299). Group composition changed on average every 16 ± 3 d when on average 6 ± 2 cows were exchanged. Cows were taken to milking twice daily at approximately 0500 and 1500 h, and fresh TMR (9% alfalfa hay, 39% corn silage, 28% grass silage, 24% concentrate and mineral mix) was delivered at approximately 0600 and 1500 h. Cows had access to water ad libitum.

Temperature and humidity measures were collected every 5 min in the pen where the group was housed using HOBO data loggers (Onset Computer Corporation, Bourne, MA). We calculated the 3-d rolling average of daily maximum THI to represent heat load (McDonald et al., 2020).

Electronic feeders and drinkers recorded the start and end times of each visit along with the ID and intake of the cow, using radiofrequency identification and a built-in scale. An algorithm was used to detect agonistic replacements based on a short time between one cow leaving and the next cow entering the same bin (Foris et al., 2019). Using the EloSteepness R package (Neumann and Fischer, 2022), we computed the summed Elo winning probability (i.e., dominance score) of cows and the hierarchy steepness based on agonistic replacements. This Bayesian-based method offers a continuous dominance evaluation in dynamic groups, estimates uncertainty, and is robust in contexts with many undefined relationships. Cows started with a prior Elo rating that was adjusted after each interaction: the cow that initiated the replacement gained points and the cow that was replaced lost points. The magnitude of change depended on the score difference between cows before the interaction, reflecting expected (smaller change in scores) versus unexpected (large change in scores) outcomes. Existing interaction history was factored in when new individuals were introduced to ensure continuity. To obtain an overall hierarchy and a steepness value for our dynamically changing group, we calculated the summed Elo winning probability (Neumann and Fischer, 2022) for each cow, a dominance measure based on their Elo rating against all potential opponents.

First, we calculated separate drinker and feeder dominance hierarchies based on 112 d of electronic bin data collected between July and December 2020. During this period, 4,021 and 82,905 replacements were detected at the drinkers and the feeders, respectively. To ensure that the amount of data used to obtain hierarchies at both resources is similar, we created a random subsample of 4,021 replacements at feeders, maintaining the original proportion of interactions for each actor cow.

Second, based on the 3-d rolling average of daily maximum THI, we divided the drinker replacement data into hot (THI ≥72; 52 d; n = 67 cows) and normal (THI <72; 60 d; n = 68 cows) periods (following McDonald et al., 2020). We then calculated separate dominance hierarchies. We determined the difference in the average daily number of replacements in the group between hot and normal periods using Welch's *t*-test.

We compared dominance hierarchies corresponding to feed or water and hot or normal periods based on their steepness. We also calculated Spearman's rank correlations between feeder and drinker hierarchies (n = 87 cows), as well as hot and normal drinker hierarchies (n = 48 cows present during both periods).

We analyzed the relationship between dominance and drinking behavior separately during hot and normal periods. In this analysis we excluded 26 d from the hot period due to detection issues with one of the water bins. We calculated the average daily value for visit frequency, time spent at the drinker, and water intake for each cow. We assessed the relationship between these variables and the Elo winning probability of cows via Spearman's rank correlations. We determined the percentages of total daily visits, total drinking duration, and total water intake during every 4-h block of the days, separately for hot and normal periods. We calculated the Spearman's rank correlation of Elo winning probability and drinking behavior percentages during each 4-h block, using the Benjamini-Hochberg correction for multiple comparisons.

We detected an average of 36 ± 10.5 replacements/d (mean ± SD) at the drinkers; this value is low compared with the average number of replacements at the feeders for the same period (740 ± 240.5 replacements/d). Accordingly, in the group of 48, on average each cow received fewer than 1 replacement/d at the drinker. In the current study stocking density was 9.6 cows/drinker. Previous work using the same type of water bin found similarly low replacement frequencies at the drinker using a stocking density of 10 cows/drinker in a group of 20 cows (Foris et al., 2019). These results confirm that the daily number of agonistic interactions at the drinker is low to reliably infer dominance, such that longitudinal recording over several days is likely needed to accurately describe the social hierarchy.

Using the replacements detected at the drinkers over time, we found differences in the individual winning probabilities of cows, suggesting the presence of dominance differences between individuals at this resource with a steepness of 0.55 ± 0.02 (Figure 1). The dominance hierarchy detected at the feeder during the same time period was less steep (steepness 0.45 ± 0.02), meaning smaller average differences between the winning probabilities of adjacently ranked individual cows when competing for feed compared with competing for water. Previous work reported higher steepness (0.97) based on all agonistic interactions observed in extensively reared beef cattle (Bagnato et al., 2023). In contrast, work using combined electronic feed and water bin data led to lower and similar steepness values in smaller groups housed under conditions similar to the current study (0.18–0.48; Foris et al., 2019). However, relying on different steepness calculation methods may also in part influence the differences between studies (de Vries et al., 2006). Taken together, these findings support the idea that cattle hierarchies under confined conditions are less steep compared with those on pasture and that the drinker-based hierarchy may be steeper than feed-based hierarchies in the context of the current study (O'Connell et al., 1989). Previous work reported that dominance influences drinking behavior when the drinker is placed in a corridor where dominants can limit access of others (Coimbra et al., 2012). In our study drinkers and feeders were installed in one row, but there were fewer drinkers with larger distances between bins, perhaps making these more likely to be monopolized by some animals. In contrast, cows may more easily access another feed bin nearby (due to a greater number of options) even during periods of high competition, making the dominant cows more willing to leave the feed bin they were using and easier to be replaced. This situation could result in a hierarchy with smaller differences in winning probabilities among cows.

**Figure 1:**
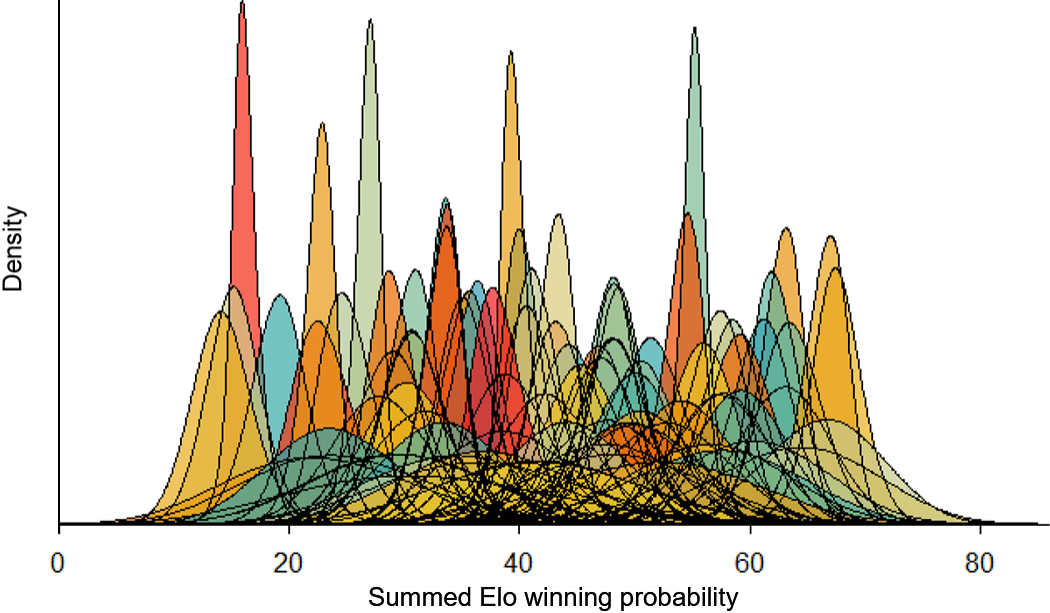
Dominance hierarchy based on automated measures of replacements at 5 drinkers in a dynamic group of 48 lactating dairy cows over 112 d. Each cow's dominance is quantified by the summed Elo winning probability on the x-axis, and density of the probability distribution of each cow on the y-axis. The density refers to probability per unit on the x-axis. More dominant individuals have a higher winning probability. A distinct color is used to represent each individual, and distributions reflect the uncertainty associated with individual winning probability estimates.

Individual cows' winning probabilities at the feeder and drinker were moderately correlated (r_s_ = 0.55, *P* < 0.001, Figure 2). In both hierarchies, we noted considerable uncertainty around the winning probability estimates of cows, especially in the middle of the hierarchy, indicating that ordinal rank may not be a meaningful representation of dominance for many members of the group. However, the most and least dominant cows showed good agreement between feeder and drinker hierarchies and a higher certainty of winning probability estimates. Dominance is considered by some as a stable individual trait (Finkemeier et al., 2018), although it is necessarily relative to other members in the group. Automatically identifying cows with consistently high or low winning probabilities could be relevant to inform grouping decisions on farms. In commercial settings, observing and recording aggressive behaviors at drinkers might be easier (and more cost effective) than doing so at a greater number of feeders to assess the social hierarchy.

**Figure 2:**
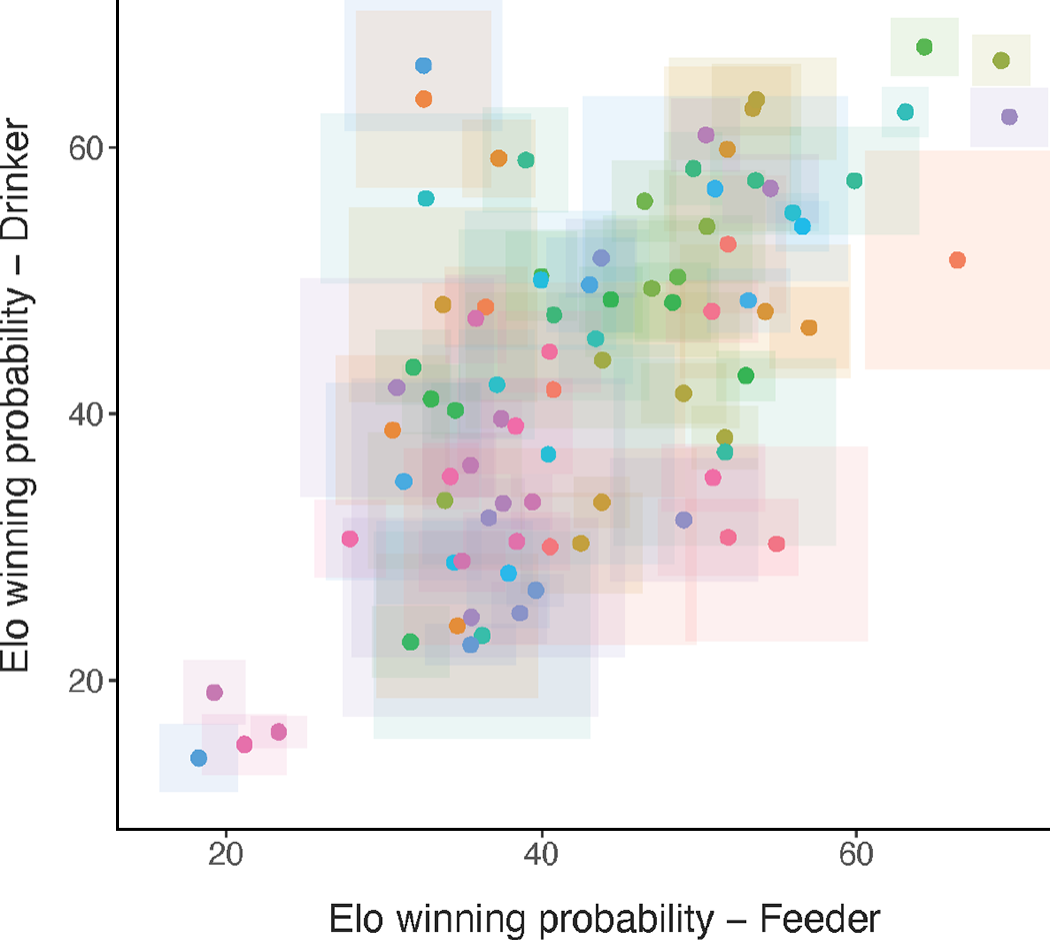
Association between social dominance (i.e., Elo winning probability) at the feeder and drinker in a dynamic group of 48 lactating dairy cows over 112 d. Points represent mean summed Elo winning probability of individual cows (n = 87), and squares around points show the associated SD. More dominant individuals have higher winning probability. A distinct color is used to represent each individual.

We investigated the influence of THI on competition for water. During hot periods the number of replacements/d averaged 39 ± 10.5 versus 34 ± 10.0 replacements/d during normal periods (*t* = 2.56, df = 105.75, *P* = 0.012). This result is consistent with previous findings for early-lactation cows, showing that hot weather increases competition at the drinker (McDonald et al., 2020). The dominance hierarchies associated with the hot and normal periods showed similar steepness (0.54 ± 0.03 vs. 0.56 ± 0.03, respectively) and the individual Elo winning probabilities of cows were highly correlated (r_s_ = 0.87, *P* < 0.001). These findings suggest that social dominance at the drinker can be stable across different THI conditions, at least under the conditions tested. It is important to note that THI fluctuates throughout the day, but we categorized replacements as either having taken place during hot or normal periods based on the 3-d rolling average of daily maximum THI. However, even during the hot period, many replacements took place during cooler times of the day. Simply summarizing results by day may obscure some of the effects of high THI on agonistic behavior at the drinker. Future research should consider THI at the hourly level, especially during longer periods of high THI, and account for other factors influencing drinking behavior (e.g., feed delivery and milking times).

When investigating the association between winning probability and average daily individual drinking behavior, we noted a weak relationship under both the hot and the normal THI conditions. Specifically, cows with a higher winning probability had lower average daily visit frequency (hot: r_s_ = −0.40, *P* < 0.01, normal: r_s_ = −0.33, *P* < 0.01) but higher average daily water intake (hot: r_s_ = 0.38, *P* < 0.01, normal: r_s_ = 0.37, *P* < 0.01). Although not investigated in this study, higher average daily water intake by more dominant cows may relate to these animals being larger, older, and higher producing (dairy, Barton, 1973; beef, Šárová et al., 2013).

The time of day when a cow chose to drink was correlated with their winning probability at the drinker. During the hot period (Figure 3A), cows with higher winning probability had a higher percentage of their daily visits to the drinker postmilking in the late afternoon and early evening hours (1600 to 2000 h; r_s_ = 0.32, *P* = 0.03). In contrast, in the late evening hours (2000 to 2400 h) a lower winning probability was associated with a higher percentage of daily visits to the drinker (r_s_ = −0.33, *P* = 0.03). During the normal period (Figure 3B), cows with a lower winning probability had a higher percentage of daily drinker visits before the morning milking (0000 to 0400 h; r_s_ = −0.32, *P* = 0.03). Cows with higher winning probability had a higher percentage of visits following the morning milking (0400 and 0800 h; r_s_ = 0.33, *P* = 0.03).

**Figure 3:**
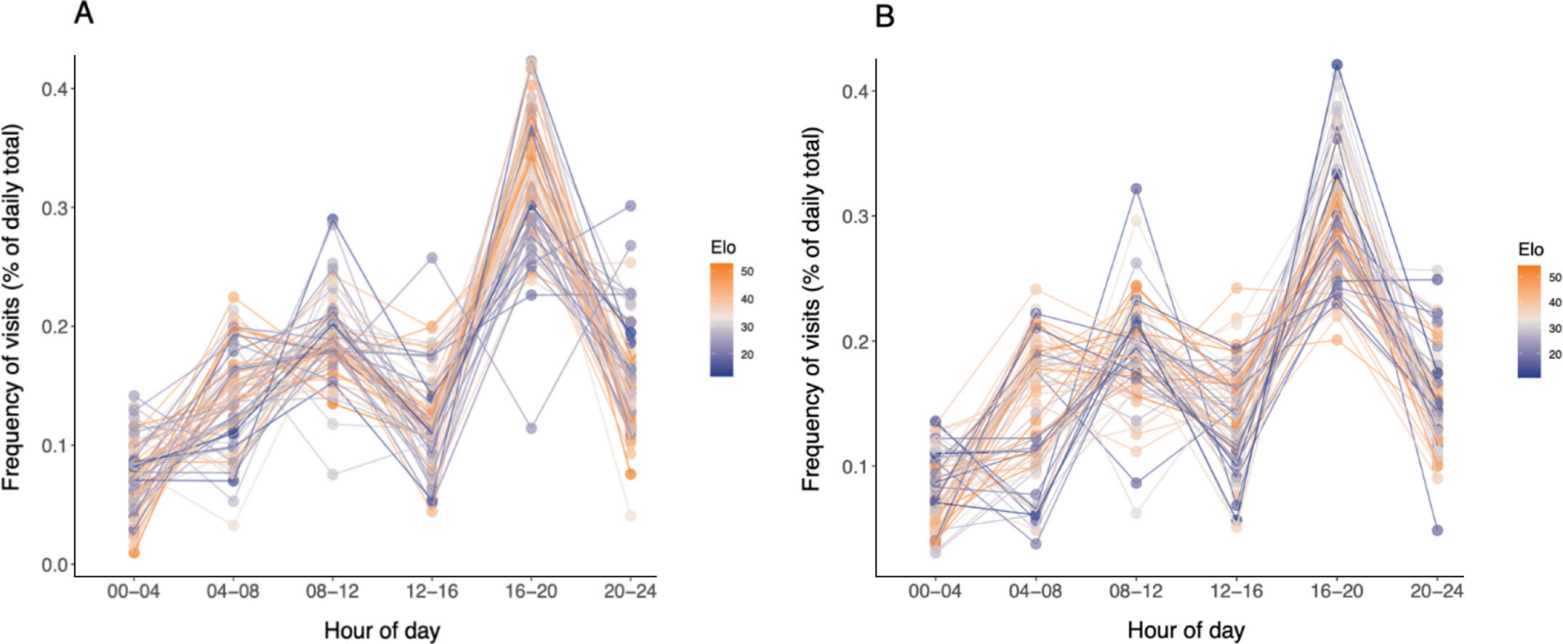
Visit frequency at the drinker during different times of the day for cows housed in a dynamic group of 48 with access to 5 electronic water bins when (A) temperature-humidity index (THI) >72 (n = 67 cows), and (B) THI <72 (n = 68 cows). Points represent average values of individual cows; color indicates social dominance measured by summed Elo winning probability at the drinker.

The similar patterns observed in the temporal distribution of visit duration and water intake suggests that the winning probability is associated with increased drinking at certain times of the day. Our results during the hot period are similar to findings reported by McDonald et al. (2020). This suggests that cows with lower winning probabilities may adjust when they drink, shifting from the postmilking visit peak to the evening when temperatures are cooler. We also found an association between drinker visit times and winning probability during the normal period. Notably, cows with higher winning probabilities spent a higher percentage of their total daily visits after the morning milking than those with a lower winning probability; these cows may have adjusted their behavior and compensated by engaging in a higher percentage of their daily visits in the early morning hours.

Given the long duration of our study, our results are more likely to be affected by fluctuations in feed composition and delivery schedules associated with normal farm practices. These factors are known to affect feeding behavior (Hosseinkhani et al., 2008; Hart et al., 2014) and thus may also affect drinking behavior. In addition, milking times coincided with feed delivery and sometimes with the hottest hours of the day, limiting our ability to draw inferences about factors causing differences in the drinking behavior of cows based on winning probability. We encourage future work to investigate the impact of different feeding regimens, milking systems (e.g., milking robots), and associated competition on drinking behavior.

## References

[bib1] Bagnato S., Pedruzzi L., Goracci J., Palagi E. (2023). The interconnection of hierarchy, affiliative behaviours, and social play shapes social dynamics in Maremmana beef cattle. Appl. Anim. Behav. Sci..

[bib2] Barton E.P., Donaldson S.L., Ross M.A., Albright J.L. (1973). Social rank and social index as related to age, body weight and milk production in dairy cows. Proceedings of the Indiana Academy of Science.

[bib3] Burkhardt F.K., Hayer J.J., Heinemann C., Steinhoff-Wagner J. (2022). Drinking behavior of dairy cows under commercial farm conditions differs depending on water trough design and cleanliness. Appl. Anim. Behav. Sci..

[bib4] Chapinal N., Veira D.M., Weary D.M., von Keyserlingk M.A.G. (2007). Technical note: Validation of a system for monitoring individual feeding and drinking behavior and intake in group-housed cattle. J. Dairy Sci..

[bib5] Coimbra P.A.D., Machado Filho L.C.P., Hötzel M.J. (2012). Effects of social dominance, water trough location and shade availability on drinking behaviour of cows on pasture. Appl. Anim. Behav. Sci..

[bib6] Crossley R.E., Harlander-Matauschek A., DeVries T.J. (2017). Variability in behavior and production among dairy cows fed under differing levels of competition. J. Dairy Sci..

[bib7] de Vries H., Stevens J.M.G., Vervaecke H. (2006). Measuring and testing the steepness of dominance hierarchies. Anim. Behav..

[bib8] Dehnen T., Arbon J.J., Farine D.R., Boogert N.J. (2022). How feedback and feed-forward mechanisms link determinants of social dominance. Biol. Rev. Camb. Philos. Soc..

[bib9] Drews C. (1993). The concept and definition of dominance in animal behaviour. Behaviour.

[bib10] Finkemeier M.-A., Langbein J., Puppe B. (2018). Personality research in mammalian farm animals: Concepts, measures, and relationship to welfare. Front. Vet. Sci..

[bib11] Foris B., Thompson A.J., von Keyserlingk M.A.G., Melzer N., Weary D.M. (2019). Automatic detection of feeding and drinking related agonistic behavior and dominance in dairy cows. J. Dairy Sci..

[bib12] Fregonesi J.A.A., Tucker C.B.B., Weary D.M.M. (2007). Overstocking reduces lying time in dairy cows. J. Dairy Sci..

[bib13] Hand J.L. (1986). Resolution of social conflicts: Dominance, egalitarianism, spheres of dominance, and game theory. Q. Rev. Biol..

[bib14] Hart K.D., McBride B.W., Duffield T.F., DeVries T.J. (2014). Effect of frequency of feed delivery on the behavior and productivity of lactating dairy cows. J. Dairy Sci..

[bib15] Hosseinkhani A., DeVries T.J., Proudfoot K.L., Valizadeh R., Veira D.M., Von Keyserlingk M.A.G. (2008). The effects of feed bunk competition on the feed sorting behavior of close-up dry cows. J. Dairy Sci..

[bib16] Jensen M.B., Vestergaard M. (2021). Invited review: Freedom from thirst—Do dairy cows and calves have sufficient access to drinking water?. J. Dairy Sci..

[bib17] Krawczel P.D., Klaiber L.B., Butzler R.E., Klaiber L.M., Dann H.M., Mooney C.S., Grant R.J. (2012). Short-term increases in stocking density affect the lying and social behavior, but not the productivity, of lactating Holstein dairy cows. J. Dairy Sci..

[bib18] McDonald P.V., von Keyserlingk M.A.G., Weary D.M. (2019). Technical note: Using an electronic drinker to monitor competition in dairy cows. J. Dairy Sci..

[bib19] McDonald P.V., von Keyserlingk M.A.G., Weary D.M. (2020). Hot weather increases competition between dairy cows at the drinker. J. Dairy Sci..

[bib20] Meyer U., Everinghoff M., Gädeken D., Flachowsky G. (2004). Investigations on the water intake of lactating dairy cows. Livest. Prod. Sci..

[bib21] Neumann C., Duboscq J., Dubuc C., Ginting A., Irwan A.M., Agil M., Widdig A., Engelhardt A. (2011). Assessing dominance hierarchies: Validation and advantages of progressive evaluation with Elo-rating. Anim. Behav..

[bib22] Neumann C., Fischer J. (2022). Extending Bayesian Elo-rating to quantify the steepness of dominance hierarchies. Methods Ecol. Evol..

[bib23] O’Connell J., Giller P.S., Meaney W. (1989). A comparison of dairy cattle behavioural patterns at pasture and during confinement. Isr. J. Agric. Res..

[bib24] Oliveira B.R., Ribas M.N., Machado F.S., Lima J.A.M., Cavalcanti L.F.L., Chizzotti M.L., Coelho S.G. (2018). Validation of a system for monitoring individual feeding and drinking behaviour and intake in young cattle. Animal.

[bib25] Pinheiro Machado Filho L.C., Teixeira D.L., Weary D.M., von Keyserlingk M.A.G., Hötzel M.J. (2004). Designing better water troughs: dairy cows prefer and drink more from larger troughs. Appl. Anim. Behav. Sci..

[bib26] Reyes F.S., Gimenez A.R., Anderson K.M., Miller-Cushon E.K., Dorea J.R., Van Os J.M.C. (2022). Impact of stationary brush quantity on brush use in group-housed dairy heifers. Animals (Basel).

[bib27] Sánchez-Tójar A., Schroeder J., Farine D.R. (2018). A practical guide for inferring reliable dominance hierarchies and estimating their uncertainty. J. Anim. Ecol..

[bib28] Šárová R., Špinka M., Stěhulová I., Ceacero F., Šimečková M., Kotrba R. (2013). Pay respect to the elders: Age, more than body mass, determines dominance in female beef cattle. Anim. Behav..

[bib29] Stoner A.W., Ottmar M.L., Hurst T.P. (2006). Temperature affects activity and feeding motivation in Pacific halibut: Implications for bait-dependent fishing. Fish. Res..

[bib30] Wierenga H.K. (1990). Social dominance in dairy cattle and the influences of housing and management. Appl. Anim. Behav. Sci..

